# Perineuronal Net Protein Neurocan Inhibits NCAM/EphA3 Repellent Signaling in GABAergic Interneurons

**DOI:** 10.1038/s41598-018-24272-8

**Published:** 2018-04-18

**Authors:** Chelsea S. Sullivan, Ingo Gotthard, Elliott V. Wyatt, Srihita Bongu, Vishwa Mohan, Richard J. Weinberg, Patricia F. Maness

**Affiliations:** 1 Department of Biochemistry and Biophysics, University of North Carolina School of Medicine, Chapel Hill, North Carolina 27599 United States; 2 Department of Cell Biology and Physiology, University of North Carolina School of Medicine, Chapel Hill, North Carolina 27599 United States

## Abstract

Perineuronal nets (PNNs) are implicated in closure of critical periods of synaptic plasticity in the brain, but the molecular mechanisms by which PNNs regulate synapse development are obscure. A receptor complex of NCAM and EphA3 mediates postnatal remodeling of inhibitory perisomatic synapses of GABAergic interneurons onto pyramidal cells in the mouse frontal cortex necessary for excitatory/inhibitory balance. Here it is shown that enzymatic removal of PNN glycosaminoglycan chains decreased the density of GABAergic perisomatic synapses in mouse organotypic cortical slice cultures. Neurocan, a key component of PNNs, was expressed in postnatal frontal cortex in apposition to perisomatic synapses of parvalbumin-positive interneurons. Polysialylated NCAM (PSA-NCAM), which is required for ephrin-dependent synapse remodeling, bound less efficiently to neurocan than mature, non-PSA-NCAM. Neurocan bound the non-polysialylated form of NCAM at the EphA3 binding site within the immunoglobulin-2 domain. Neurocan inhibited NCAM/EphA3 association, membrane clustering of NCAM/EphA3 in cortical interneuron axons, EphA3 kinase activation, and ephrin-A5-induced growth cone collapse. These studies delineate a novel mechanism wherein neurocan inhibits NCAM/EphA3 signaling and axonal repulsion, which may terminate postnatal remodeling of interneuron axons to stabilize perisomatic synapses *in vivo*.

## Introduction

A correct balance of excitatory and inhibitory (E/I) connectivity in cortical networks is established postnatally through concurrent processes of synaptogenesis and synaptic remodeling. Alterations in the density of inhibitory synapses can affect circuit function and are linked with cognitive dysfunction in schizophrenia, autism and other neurological disorders^1^. The perisomatic region (soma and proximal dendrites) of excitatory pyramidal neurons is targeted principally by inhibitory basket interneurons^2^. During postnatal development of the neocortex, parvalbumin-positive (PV^+^) basket cells form excess numbers of perisomatic synapses, which are subsequently eliminated in substantial numbers upon maturation to obtain an optimal density^3^. In the prefrontal cortex, basket interneurons are vital in regulating action potential firing and synchrony of pyramidal neurons important for cognitive processes such as working memory^4^.

A key unanswered question is how basket cell synaptic remodeling is terminated upon maturation. Perineuronal nets (PNNs) are potential candidates for restricting the remodeling of GABAergic terminals and thus regulating perisomatic synapse density. These extracellular matrix components coalesce through interactions among chondroitin sulfate proteoglycans (CSPGs), which contain glycosaminoglycan chains (GAGs) and hyaluronic acid, to form lattice-like structures around neuronal soma and proximal dendrites^5–8^. As shown in visual cortex^9^, amygdala^10^, and hippocampus^11^, formation of PNNs during maturation restricts plasticity, whereas removal of these nets by enzymatic digestion of GAG chains reinstates juvenile-like levels of synapse remodeling. Defects in PNNs have been associated with neurological disorders including schizophrenia, bipolar disorder, autism spectrum disorders, and major depression^12,13^. Although PNNs are associated with restriction of synaptic plasticity, little is known about the molecular mechanism of PNNs in regulating synaptic maturation.

The mouse medial frontal cortex (referred to as PFC hereafter), is considered to share overlapping functional homology with human prefrontal cortex, including regulation of working memory and social function^14^. Ephrin-A repellent ligands in the mouse PFC engage a receptor complex consisting of the tyrosine kinase EphA3 and immunoglobulin (Ig)-class cell adhesion molecule NCAM, which signals within GABAergic axon terminals to constrain the density of basket cell synapses at the perisomatic region of pyramidal cells^15,16^. Deletion of NCAM, EphA3, or ephrin-A2,-A3,-A5 in mice increases the density of perisomatic basket cell synapses^16^, consistent with a role for NCAM and EphA3 in ephrin-A-induced synaptic remodeling. In accord with the increased density of somatically-targeted inhibitory synapses in NCAM null mice, optogenetic studies further demonstrated that NCAM deletion increases functional GABAergic connectivity with pyramidal neurons in PFC (layers 2,3)^17^.

Neurocan is a CSPG that functions as a pivotal organizer of PNNs, and is a potential risk factor for schizophrenia^18,19^ and bipolar disorder^20^. To investigate a role for neurocan as a regulator of ephrinA5-induced perisomatic synapse remodeling, we investigated its ability to perturb the functional interactions of NCAM and EphA3 in the developing mouse PFC. To determine if neurocan regulates ephrinA5-induced perisomatic synapse remodeling, we investigated its ability to perturb the functional interactions of NCAM and EphA3 in the developing mouse PFC. Enzymatic degradation of PNN components to release GAG chains decreased perisomatic synapse density in organotypic slices from PFC and disrupted NCAM/EphA3 association. Moreover, neurocan inhibited ephrinA5-induced clustering of NCAM and EphA3, impairing EphA3 kinase activation and GABAergic axon repulsion. These results delineate a novel mechanism by which neurocan-containing PNNs terminate postnatal remodeling of basket cell terminals by inhibiting the interaction of NCAM with EphA3 in the PFC.

## Results

### Degradation of PNN GAG Chains Reduces Perisomatic Synapse Density in Cortical Slices

**Figure 1:**
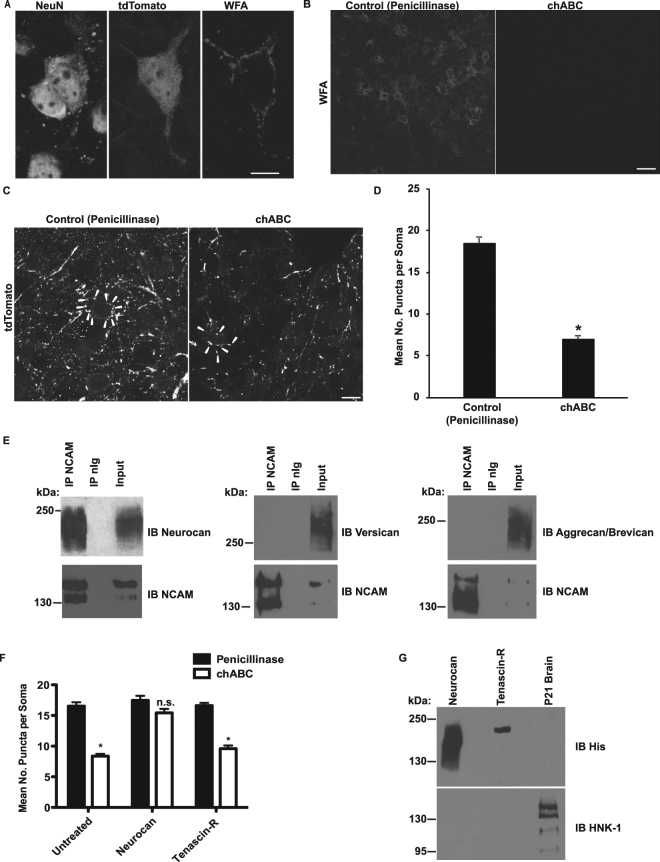
GAG-modified neurocan blocks chABC-induced decrease of perisomatic synaptic puncta in organotypic brain slices. (**A**) Immunostaining of neuronal soma (NeuN), a parvalbumin-positive interneuron (tdTomato), and a perineuronal net (WFA) in DIV14 organotypic brain slice culture. Scale bar = 10 μm. (**B**) WFA labeling of perineuronal nets in control penicillinase and chABC-treated brain slices. Scale bar = 30 μm. (**C**) Representative image of perisomatic synapses (tdTomato) in control penicillinase or chABC-treated slice cultures. Representative perisomatic puncta around a single soma are indicated with arrowheads. Scale bar = 10 μm. (**D**) Quantification of the mean number of perisomatic synaptic puncta per soma (n = 30 soma/condition, 3 animals per condition, t-test, *p < 0.05). (**E**) NCAM was immunoprecipitated from brain lysates, followed by immunoblotting with antibodies against neurocan, versican, or aggrecan/brevican (using an antibody raised against shared epitope). (**F**) Slices were treated with control penicillinase or chABC as in (**C**) followed by rescue with neurocan or tenascin-R. Quantification of the mean number of perisomatic synapses per soma was performed (>90 soma per mouse per condition, n = 3 mice, two-way ANOVA with Bonferonni post-hoc testing, *p < 0.05). (**G**) Immunoblot to detect recombinant proteins (immunoblotted for His tag) and HNK-1 carbohydrate modification. P21 brain lysate was used as a positive control for HNK-1 signal.

To directly test the importance of PNN molecules in regulation of perisomatic GABAergic synapse density in the PFC, organotypic slice cultures were prepared from Parvalbumin-Cre;Ai9 (Lox-STOP-Lox tdTomato) mice, in which parvalbumin-positive interneurons express tdTomato throughout the soma and neuronal processes as a reporter of Cre-dependent recombination^21,22^. Interneurons in the PFC in these slice cultures form characteristic axon arbors and perisomatic boutons over 14 days *in vitro* (DIV)^16^ with a time course analogous to that observed *in vivo*^23^. We focused on the anterior cingulate region of the PFC (layers 2,3), because it is a region where synaptic plasticity is pronounced and intra- and subcortical inputs are maximal^24,25^. *Wisteria Floribunda* agglutinin (WFA) is a lectin that binds to residues found within PNN GAG chains^26^. WFA labeling of PNNs in layers 2,3 of the anterior cingulate at 14 DIV showed characteristic PNNs around populations of NeuN-positive neuronal soma, as well as tdTomato-labeled interneurons (Fig. 1A).

To determine if GAG-modified PNNs restrained postnatal remodeling of perisomatic basket cell synapses, slice cultures (DIV12) were treated with a control enzyme (penicillinase) or the bacterial enzyme chondroitinase ABC (chABC) for 2 hours, followed by removal of enzyme and further incubation until fixation at DIV14 (equivalent postnatal day 19). Penicillinase is a bacterial-derived enzyme that is used as a non-relevant control for comparison to chABC^27^. chABC treatment was sufficient to remove PNNs as indicated by a loss of WFA staining in the chABC treated slices (Fig. 1B). Treatment with chABC significantly decreased the mean number of perisomatic puncta per soma in comparison to control penicillinase, implicating PNN proteins as a stabilizing factor for perisomatic synapses (Fig. 1C,D). Neurocan coimmunoprecipitated with NCAM in brain lysates (Fig. 1E), and previous work indicates that purified NCAM and neurocan can associate in a partially GAG-dependent manner *in vitro*^28^. Chondroitinase ABC will target the GAG chains of neurocan and other CSPGs in PNNs. However, NCAM did not bind other CSPG proteins (versican, aggrecan, brevican), as shown by lack of co-immunoprecipitation from brain lysates (Fig. 1E). Although NCAM can bind the CSPG phosphacan, this depends on the core protein rather than GAG chains^29^.

Although our chABC results in organotypic slices suggested that PNN components may regulate perisomatic synapses, no specific target of chABC in this process was identified. We hypothesized that neurocan may regulate perisomatic synapses based on its binding to NCAM. However, chABC treatment has also been shown to reduce tenascin-R^6^, a PNN glycoprotein that contributes to formation and activity of perisomatic GABAergic interneurons in the CA1 area of the hippocampus^30–33^. Thus, we wanted to identify whether neurocan and/or tenascin-R were involved in setting the number of perisomatic GABAergic synapses in organotypic slices. To assess the role of these proteins in regulation of perisomatic synapses, we performed a rescue experiment in which organotypic slices were treated with control penicillinase or chABC (DIV 10, 2 hours) followed by removal of enzyme and addition of no protein (untreated), neurocan, or tenascin-R. Rescue with recombinant neurocan but not tenascin-R prevented the decrease of perisomatic synapses previously observed after chABC treatment (Fig. 1F). However, further testing of the recombinant tenascin-R protein for the presence of HNK-1 carbohydrate modification indicated that the protein lacked detectable HNK-1 (Fig. 1G). HNK-1 modification has been shown to be required for the synaptogenic effects of tenascin-R^30–33^. The rescue experiment with recombinant neurocan indicates that neurocan but not tenascin-R lacking HNK-1 is sufficient to rescue perisomatic innervation after chABC.

### Expression of Neurocan and NCAM in the Postnatally Developing PFC

**Figure 2:**
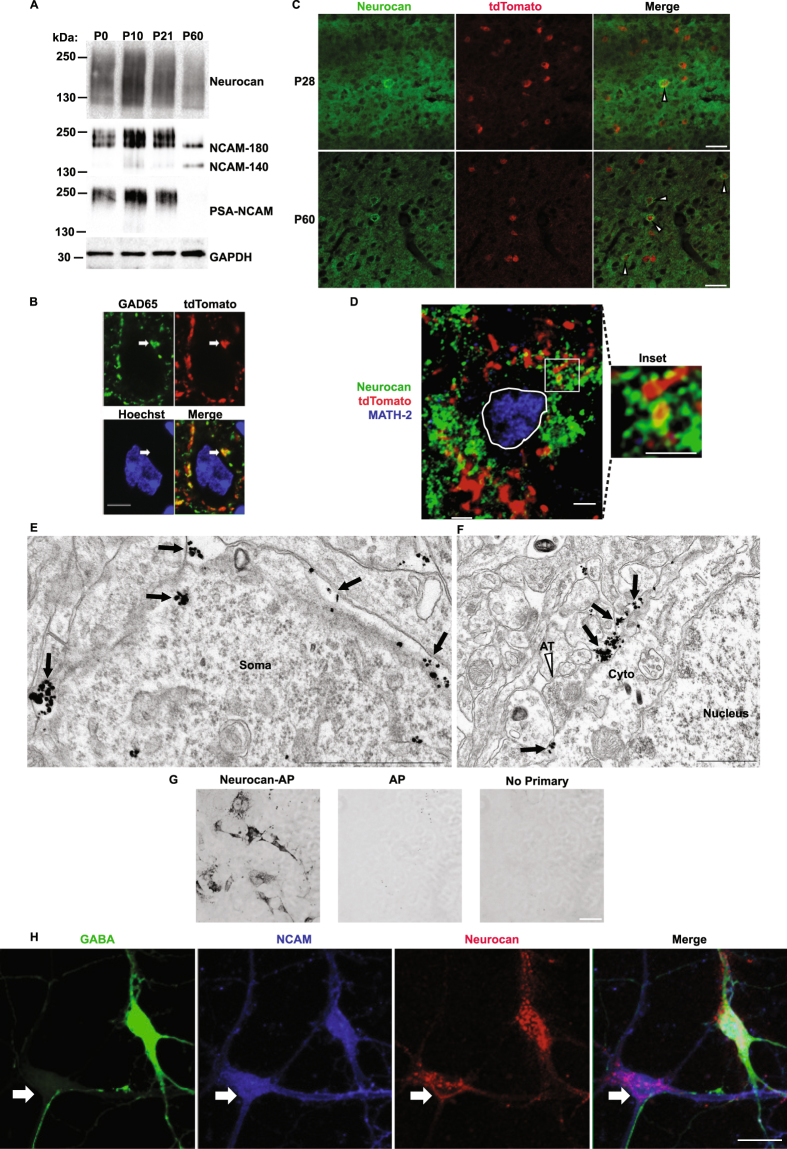
Expression of neurocan and NCAM in postnatal and adult brain. (**A**) Immunoblot of total brain extracts for neurocan, NCAM, PSA-NCAM, and GAPDH (loading control). (**B**) Co-localization of tdTomato puncta (red) with the presynaptic marker GAD65 (green) around a neuronal soma (blue). Scale bar = 5 μm. (**C**) Immunofluorescent staining of neurocan (green) around PV^+^ tdTomato-labeled interneurons (red) in mouse frontal cortex at P28 and P60. Scale bar = 50 μm. (**D**) Confocal images of neurocan (green) around perisomatic synaptic puncta (red) onto MATH-2 expressing pyramidal neurons (blue nucleus, outlined with white border). A magnified inset is indicated with a white box. Scale bars = 2.5 μm. (**E**) Electron micrograph of immunogold labeling for neurocan along the membrane of a neuronal soma (black arrows point to examples). Scale bar = 1 μm. (**F**) Electron micrograph of an inhibitory axon terminal onto a neuronal soma. Neurocan immunogold labeling is indicated with black arrows, and the axon terminal (AT) is labeled with a white arrowhead. Cytoplasm and nucleus of the soma are identified with text. Scale bar = 1 μm. (**G**) Verification of neurocan antibody specificity by immunostaining of COS7 cells transfected with Neurocan-AP (+control) or AP (−control), and a no primary antibody control. Scale bar = 50 μm.(**H**) Immunofluorescent localization of NCAM and neurocan in GABA+ and GABA− cortical neuron cultures. Confocal images of GABA (green), NCAM (blue), and neurocan (red) in cortical neuron cultures. White arrow indicates a GABA-negative neuron positive for NCAM and neurocan, and the green neuron represents a GABAergic interneuron. Scale bar = 10 μm.

The temporal and spatial expression of neurocan in the neocortex could affect its function in specific cell types or brain circuits. Neurocan was found to be prominently expressed in mouse brain at birth (postnatal day zero, P0), increased at early postnatal and adolescent stages (P10-P21), and decreased at adulthood (P60) (Fig. 2A), similar to previous results in rat brain^34^. Neurocan was observed as a diffuse protein (130–250 kDa) on immunoblots due to GAG chain modification. NCAM exhibited a similar developmental pattern of expression as neurocan and displayed regulated changes in polysialylation. NCAM was evident at P0-P21 as a high molecular weight (180–250 kDa) diffuse species that was recognized by PSA-specific antibodies (Fig. 2A). In young adults at P60 NCAM resolved into non-PSA NCAM isoforms of 180- and 140-kDa, which differ only in the length of their cytoplasmic domains^35^ (Fig. 2A).

The cellular localization of neurocan in the PFC was next evaluated by immunofluorescence staining in Parvalbumin-Cre;Ai9 reporter mice at P28 (immature) and P60 (adult) (Fig. 2B). In the PFC of these mice, perisomatic puncta of TdTomato-positive basket cells co-localized with GAD65, a presynaptic marker enriched in GABAergic nerve terminals (Fig. 2B). Neurocan was abundant and diffusely localized throughout PFC laminae at P28, and decreased by P60 (Fig. 2C). Somata of many tdTomato-positive interneurons were distinctly outlined by neurocan immunofluorescence (9% and 17% at P28 and P60, respectively) (Fig. 2C). Neurocan localization around pyramidal cells was validated by double immunostaining with the nuclear transcription factor MATH-2, which is specific for pyramidal neurons (Fig. 2D)^36^. Neurocan staining was observed within approximately 1 μm of 81% of tdTomato-positive perisomatic puncta around MATH-2-expressing pyramidal cells (Fig. 2D) (n = 3 mice, 150 tdTomato puncta, SEM = 6%). Neurocan localization in the PFC (P18) was further probed at the electron microscope level by immunogold labeling. Neurocan labeling was observed at discrete sites along the somal membrane as well as in the extracellular space adjacent to soma (arrows, Fig. 2E). Neurocan labeling was sometimes seen at the somal membrane in proximity to synaptic vesicle-containing axon terminals (AT, open arrowhead; Fig. 2F), but was rarely seen within the cytoplasm. To verify the specificity of the neurocan antibody, COS7 cells were transfected to express neurocan fused to alkaline phosphatase (Neurocan-AP), or alkaline phosphatase (AP) alone. Fixed cells were subjected to indirect immunoperoxidase staining with or without neurocan antibodies (Fig. 2G). Only cells expressing Neurocan-AP were positive for labeling. The AP transfected cells and control lacking primary antibody showed no signal, indicating specificity of the antibody. To assess the spatial relationship of neurocan and NCAM in both pyramidal neurons and inhibitory GABAergic interneurons, cortical neuron cultures (14DIV) were stained with antibodies against GABA, NCAM, and neurocan (Fig. 2H). Results indicated that both GABA-positive interneurons and GABA-negative neurons express NCAM and neurocan at neuronal soma (Fig. 2H). Together, these results showed that neurocan was preferentially localized in postnatally developing PFC near neuronal soma membranes and was present around both excitatory and inhibitory neurons.

### NCAM Polysialylation Inhibits Binding to Neurocan

**Figure 3:**
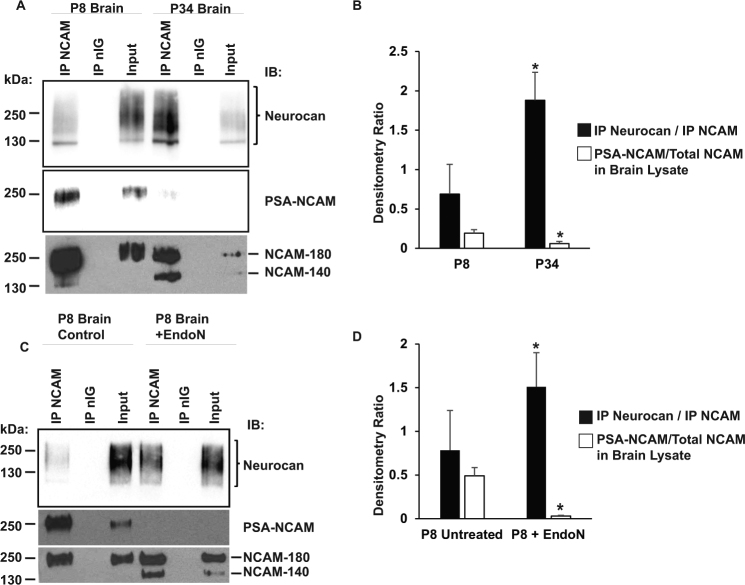
PSA inhibits binding of NCAM to neurocan. (**A**) Co-immunoprecipitation of neurocan and NCAM from P8 or P34 brain extracts immunoblotted for neurocan, NCAM, and PSA-NCAM. (**B**) Densitometry of (**A**). Graph indicates the ratio of bound neurocan to immunoprecipitated NCAM using black bars and ratio of PSA-NCAM to total NCAM in brain lysate (white bars) (n = 3, t-test, *p < 0.05). (**C**) Co-immunoprecipitation of neurocan and NCAM from untreated or EndoN-treated brain extracts immunoblotted for neurocan, NCAM, and PSA-NCAM. (**D**) Densitometry of (**C**). Graph indicates the ratio of bound neurocan to immunoprecipitated NCAM (black bars) and ratio of PSA-NCAM to total NCAM in brain lysate (white bars) (n = 3, t-test, *p < 0.05).

Polysialylation of NCAM is required for ephrinA-induced repellent responses of GABAergic interneurons^16^. To determine whether neurocan binds differentially to polysialylated NCAM or unmodified NCAM isoforms, NCAM and neurocan association was analyzed by co-immunoprecipitation from brain lysates at P8, when NCAM is heavily polysialylated, and at P34, when only a small portion of NCAM is PSA-modified (Fig. 3A). Neurocan preferentially immunoprecipitated with non-PSA NCAM at P34 compared to PSA-NCAM at P8 (Fig. 3A,B). To determine if PSA removal increased NCAM/neurocan binding, P8 brain lysates were treated with or without endoneuraminidase N (EndoN) to remove PSA, followed by immunoprecipitation of NCAM. EndoN treatment was effective at decreasing NCAM polysialylation, and significantly increased neurocan binding to non-PSA NCAM-180 and NCAM-140 (Fig. 3C,D). These results support the conclusion that neurocan preferentially binds non-PSA NCAM isoforms.

### Neurocan Binds to the Ig2 Domain of NCAM

**Figure 4:**
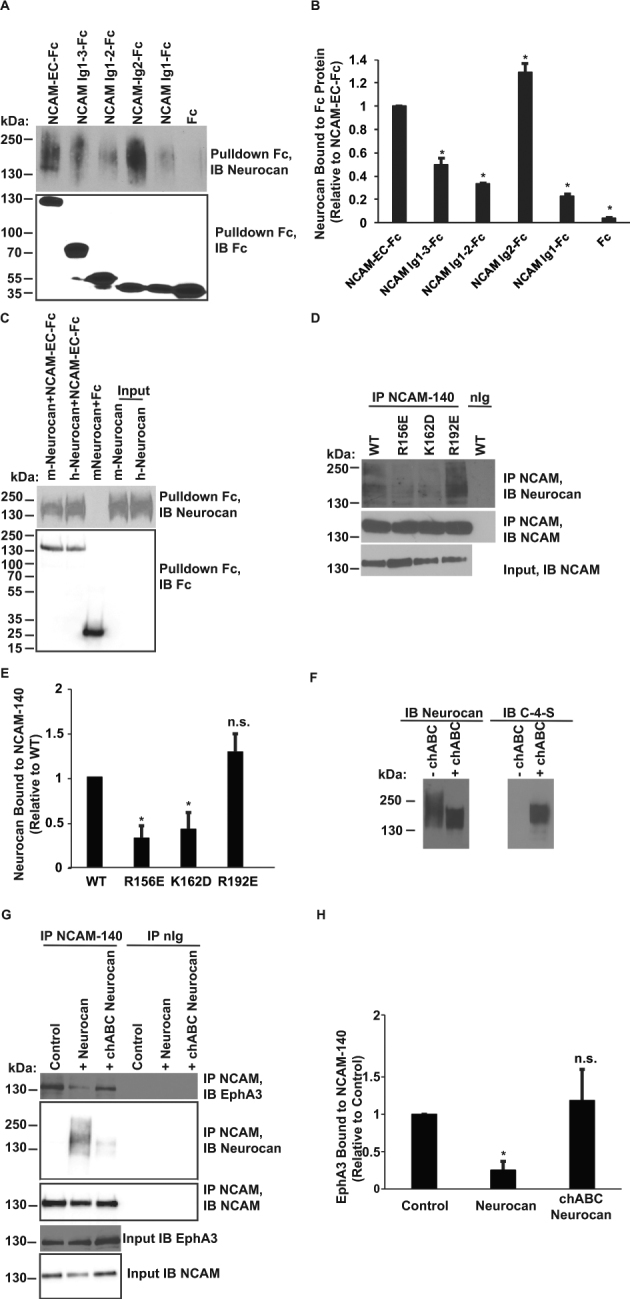
Neurocan binds the Ig2 domain of NCAM, decreasing EphA3 binding. (**A**) Fc-pulldowns of the NCAM extracellular domain (NCAM-EC), truncation mutants of NCAM, or control Fc and recombinant neurocan. (**B**) Densitometry of (**A**) indicating the level of neurocan bound (relative to positive control NCAM-EC-Fc bound neurocan) for each construct (*p < 0.05 compared to NCAM-EC-Fc) (C) Fc-pulldowns of NCAM-EC-Fc or control Fc with mouse neurocan (lacking sushi domain) and full-length human neurocan. (**D**) Co-immunoprecipitation of WT NCAM-140 or mutants of NCAM and neurocan from transfected HEK293T cells. (**E**) Densitometry of (**D**). The amount of co-immunoprecipitated neurocan for each NCAM IP was normalized to control WT NCAM-bound neurocan (n = 3, t-test, *p < 0.05). (**F**) Immunoblot of untreated or chABC-treated neurocan protein probed for neurocan or C-4-S. (**G**) Co-immunoprecipitation of NCAM-140 and EphA3 from transfected HEK293 cells treated with no neurocan (control), neurocan, or chABC-treated neurocan. (**H**) Densitometry of (**G**). The amount of co-immunoprecipitated EphA3 for each NCAM IP was normalized to control NCAM-bound EphA3 (n = 3, t-test, *p < 0.05).

NCAM contains a heparin sulfate proteoglycan (HSPG) binding site in its Ig2 domain^37^, and residues within this proteoglycan binding domain (R156 and K162) are required for EphA3 binding^38^. Because the CSPG neurocan and HSPGs are extensively modified by GAG chain addition, it was hypothesized that neurocan engages the NCAM extracellular region (NCAM-EC) within the Ig2 proteoglycan binding domain. The neurocan binding site on NCAM was identified using assays in which Fc fusion proteins consisting of human NCAM Ig1-5/FN1-2 (full-length NCAM-EC), Ig1-3, Ig1-2, Ig2, and Ig1 (or Fc alone as a control) were incubated with recombinant human neurocan, and then pulled down with Protein A/G-Sepharose beads. Results showed that neurocan bound most effectively to full-length NCAM-EC and NCAM Ig2 (Fig. 4A,B). Neurocan bound to a lesser extent to NCAM truncation fragments Ig1-3, Ig 1-2, and Ig1 (50%, 33%, and 23% compared to NCAM-EC-Fc binding, respectively) (Fig. 4A,B). Quantification of binding showed that neurocan binding to NCAM Ig2-Fc was greater than to NCAM-EC (129% compared to amount of neurocan bound to NCAM-EC-Fc) (Fig. 4A,B). Apart from Ig1-Fc, all other constructs tested contained the Ig2 domain but bound with varying affinities to neurocan. Structural studies suggest that NCAM forms dimers through *cis* interactions between its Ig1 and Ig2 domains^39^ and also *trans* interactions involving Ig3. The presence or absence of various Ig domains may influence the ability of NCAM to dimerize in solution, affecting accessibility of the Ig2 binding site of neurocan and potentially resulting in different affinities for neurocan binding. As the NCAM Ig2-Fc fragment is free from dimer-induced steric hindrance by Ig1 or Ig3, it may be more accessible for neurocan binding, which is consistent with the binding results.

Neurocan contains a carboxyl terminal sushi domain, a structure known to mediate protein-protein interactions. NCAM binds EphA3 at its cysteine-rich-domain (CRD), which also contains a sushi domain^38^. The Ig family member, L1-CAM, interacts with the sushi domain of neurocan^40^. Thus, it was hypothesized that the sushi domain of neurocan might be involved in NCAM/neurocan binding. The contribution of the neurocan sushi domain to NCAM binding was tested using recombinant human neurocan (full-length protein) or mouse neurocan Glu23-637 (a truncation lacking the sushi domain). These recombinant neurocan proteins were incubated with human NCAM-EC-Fc, and the amount of bound neurocan was assessed after Fc-pulldown. Both mouse and human neurocan bound efficiently to NCAM-EC-Fc. Binding of mouse neurocan lacking the sushi domain was somewhat decreased compared to full-length neurocan (93% relative to human neurocan level bound to NCAM), but this difference was not statistically significant (p = 0.23, t-test) (Fig. 4C).

A complementary charged interface between the extracellular NCAM Ig2 domain and the EphA3 CRD mediates the binding and clustering of NCAM and EphA3 in the neuronal membrane. Amino acids R156 and K162 in the NCAM Ig2 domain were identified by molecular modeling and determined to be important residues for NCAM binding to EphA3^38^. These residues are also part of a domain required for NCAM binding to heparin sulfate proteoglycans (HSPGs)^39^. In contrast, another Ig2 residue (NCAM R192) is not within the EphA3 binding site^38^. To assess the contribution of residues in NCAM Ig2 to neurocan binding, lysates of HEK293T cells expressing wild type (WT) NCAM or NCAM mutants (R156E, K162D, and R192E) were incubated with full-length neurocan, and the amount of neurocan co-immunoprecipitating with NCAM was analyzed by immunoblotting. Neurocan co-immunoprecipitated with WT NCAM and NCAM R192E, but bound to a much lesser extent with NCAM R156E and K162D mutants (32% and 41% of WT) (Fig. 4D,E, p < 0.05). These results indicate that neurocan binding to NCAM Ig2 involves residues involved in EphA3 association and HSPG binding.

### Neurocan GAG Chains Mediate NCAM Binding and Disrupt the NCAM/EphA3 Interaction

The shared NCAM binding site of neurocan and EphA3 led us to the hypothesis that competitive binding may occur between these proteins. To assess the ability of neurocan to disrupt NCAM/EphA3 binding and to test the importance of GAG chains in this process, a cell-culture binding assay was developed. Conditions were established to pre-treat recombinant neurocan with chABC followed by heat inactivation of enzyme. The efficiency of GAG removal was confirmed by a loss of high molecular weight GAG-modified neurocan by SDS-PAGE (Fig. 4F). Further confirmation of chABC efficacy was obtained by immunoblotting for chondroitin-4-sulfate (C-4-S) stubs which are generated after chABC treatment. C-4-S signal was observed after chABC treatment but not in control sample processed without enzyme, indicating that chABC treatment was effective (Fig. 4F). HEK293T cells expressing non-PSA NCAM-140 and EphA3 were treated with neurocan, chABC-pretreated neurocan, or left untreated prior to lysis. NCAM-140 was immunoprecipitated, and the amounts of bound EphA3 and neurocan were analyzed by immunoblotting. EphA3 binding was significantly decreased to a level of 25% of control in neurocan-treated cells (p < 0.05), but chABC-treated neurocan did not significantly alter EphA3 binding (p > 0.05) (Fig. 4G,H). Furthermore, after chABC treatment, 69% less neurocan was bound to non-PSA NCAM-140 compared to control neurocan (Fig. 4G, p < 0.05). These results indicated that neurocan decreased binding of EphA3 to non-PSA NCAM, and that neurocan/NCAM binding required intact GAG chains of neurocan.

### Neurocan Inhibits Ephrin-A5-induced Clustering/Activation of NCAM and EphA3 in Cultured Cortical Interneurons

**Figure 5:**
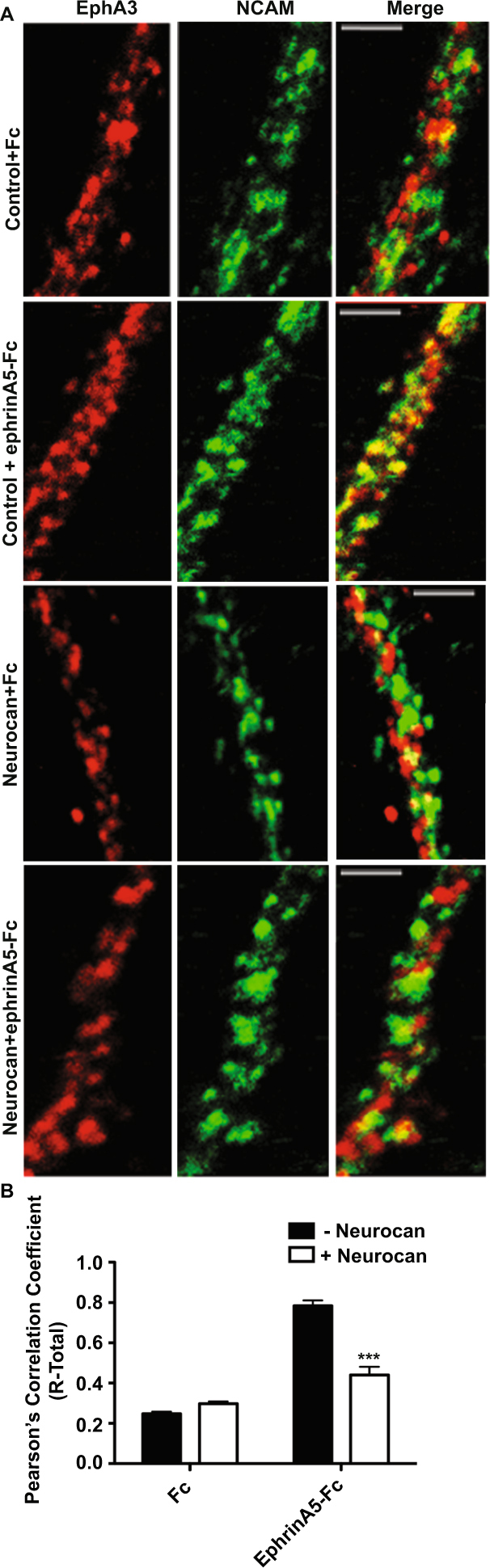
Neurocan impairs ephrin-A5-mediated clustering of NCAM and EphA3 in cortical interneurons in culture. (**A**) Cortical neuron cultures were pretreated with no neurocan (control) or neurocan followed by preclustered Fc or ephrin-A5-Fc, and localization of endogenous NCAM (green) and EphA3 (red) was assessed in axons of GABA immunopositive axons by confocal microscopy. Scale bars = 5 μM. (**B**) Pearson’s Correlation Coefficients (R-Total) were generated for each condition using ImageJ co-localization software (n = 3, two-way ANOVA with Bonferonni post-hoc testing, *p < 0.05).

The activation of Eph receptor signaling is initiated by ephrin-induced receptor oligomerization in the plasma membrane^41^. Mechanistically, NCAM promotes ephrin-A-induced repulsion in GABAergic neurons by stimulating EphA3 receptor clustering, tyrosine kinase activation, and signaling through the small GTPase RhoA^38^. NCAM has been shown to co-cluster with EphA3 and to potentiate oligomerization in processes of GABAergic interneurons in cortical cultures^38^. The effect of neurocan on ephrin-A5-induced clustering of endogenous NCAM and EphA3 was assessed in mouse cortical cultures using this assay^38^. Neuronal cultures (14 DIV) were incubated with or without recombinant neurocan (4 nM, 30 minutes) before stimulation with control Fc or ephrin-A5-Fc for 30 minutes. Immunostaining of NCAM, EphA3, and GABA (to identify interneurons) was performed after cell fixation. Co-clustering of NCAM and EphA3 was analyzed in confocal images in which only GABA-labeled regions of interest (ROIs) were selected using ImageJ co-localization software. Results of the analysis yielded Pearson’s Correlation Coefficients (R-Total), ranging from −1 (no correlation) to 1 (absolute correlation). In untreated cells, ephrin-A5-Fc induced strong co-localization of NCAM and EphA3 as seen in merged images and a 4-fold increase in R-Total (Fig. 5A,B). In contrast, ephrin-A5-induced co-localization of NCAM and EphA3 in neurocan-treated cells was significantly reduced (Fig. 5A,B). These findings show that neurocan can block NCAM/EphA3 co-clustering on GABAergic neuronal processes, in accord with its ability to perturb binding.

**Figure 6:**
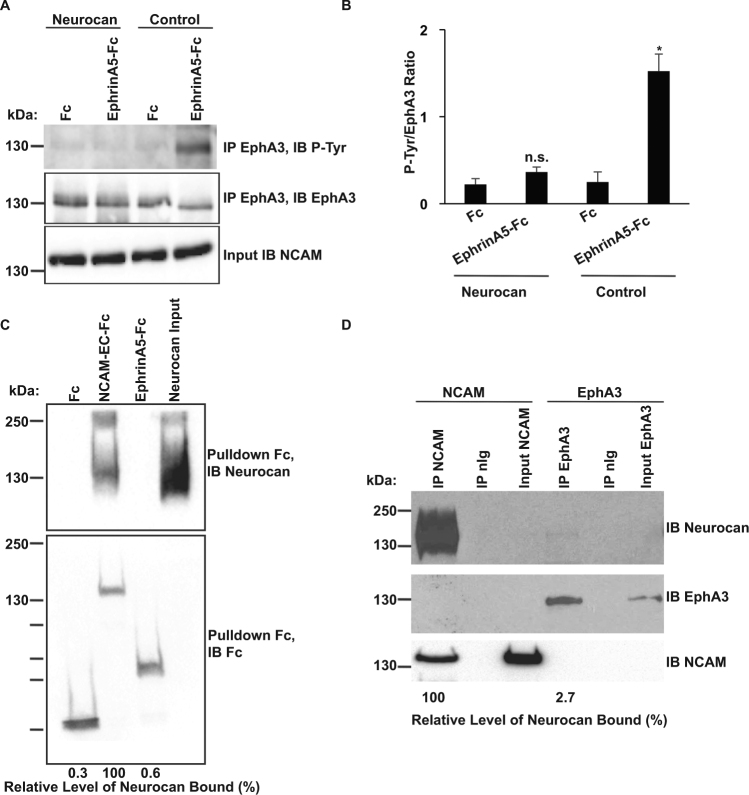
Neurocan decreases ephrin-A5-induced EphA3 autophosphorylation. (**A**) HEK293T cells transfected with NCAM and EphA3 were treated with preclustered control Fc or ephrin-A5-Fc, and EphA3 was immunoprecipitated. EphA3 autophosphorylation was assessed by immunoblotting with a phosphotyrosine antibody (PY99). Total levels of immunoprecipitated EphA3 were assessed by reprobing with EphA3 antibody. (**B**) Densitometry of (**A**). Graph indicates the ratio of phosphotyrosine to EphA3 values for each condition (n = 3, *p < 0.05). (**C**) Fc-pulldowns of control Fc, NCAM-EC-Fc, and ephrin-A5-Fc with recombinant neurocan. Level of neurocan bound (relative to positive control NCAM-EC-Fc bound neurocan) is indicated as a percentage under each lane. (**D**) Co-immunoprecipitation of NCAM (positive control) or EphA3 with neurocan from transfected HEK293T cells. Level of neurocan bound (relative to positive control NCAM-EC-Fc bound neurocan) is indicated as a percentage under each lane.

Ephrin-A-induced clustering of EphA receptors induces autophosphorylation of tyrosine residues in the receptor cytoplasmic domain^42,43^ leading to downstream signaling. To evaluate the consequences of neurocan on EphA3 kinase activation, EphA3 autophosphorylation was assessed in HEK293T cells co-expressing NCAM-140 and EphA3. Cells were treated with or without neurocan (20 nM, 30 minutes), then stimulated with control Fc or ephrin-A5-Fc. EphA3 was immunoprecipitated and immunoblotted with phosphotyrosine antibody (PY99), followed by stripping and reprobing for EphA3 protein. The ratio of phospho-EphA3 to total EphA3 was quantified by densitometry and compared for significant differences. In control cultures treated with ephrin-A5-Fc, EphA3 phosphorylation was strongly increased in comparison to Fc treated cells (Fig. 6A,B). In neurocan-treated cells, ephrin-A5-induced EphA3 autophosphorylation was effectively blocked (Fig. 6A,B). Binding of neurocan to other components of the NCAM/EphA3/ephrin-A5 signaling complex was also assessed. In pull down assays, purified recombinant neurocan bound to NCAM-EC-Fc but did not bind ephrin-A5-Fc (relative level of neurocan bound to EphrinA5-Fc was 0.6% of neurocan bound to NCAM-EC-Fc, n = 3, p < 0.05) (Fig. 6C). Results of co-immunoprecipitation from HEK293T cells expressing NCAM140 or EphA3 indicated that neurocan bound to NCAM but not to EphA3 (relative level of neurocan bound to EphA3 was 2.7% of neurocan bound to NCAM, n = 3, p < 0.05) (Fig. 6). Together, these results indicated that neurocan engages NCAM at the same extracellular motif required for EphA3 binding, inhibiting ephrin-A5-induced receptor clustering and EphA3 receptor activation.

### Neurocan Perturbs Ephrin-A5-induced Growth Cone Collapse of GABAergic Interneurons

**Figure 7:**
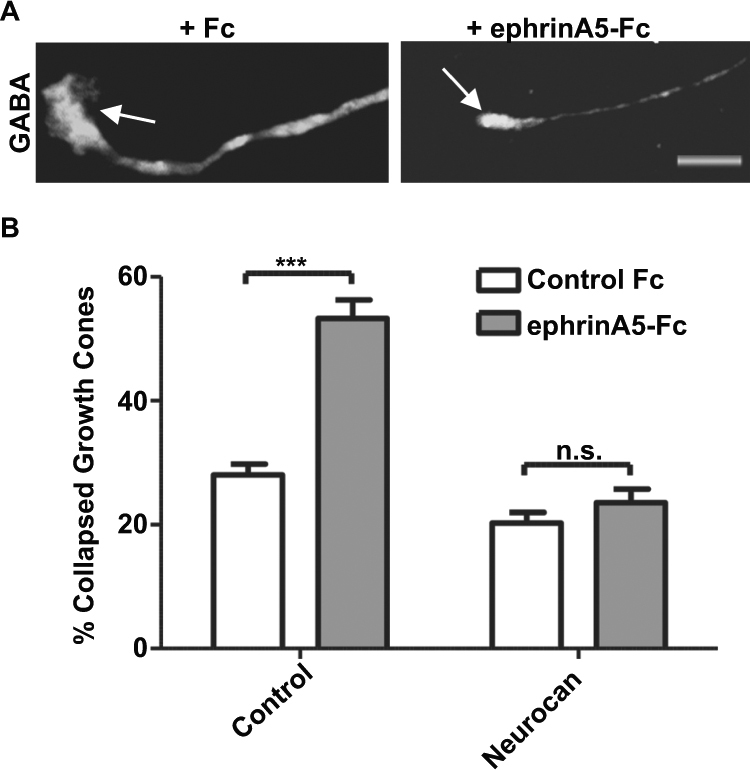
Neurocan inhibits ephrin-A5-induced growth cone collapse in GABAergic interneurons. (**A**) Representative spread and collapsed growth cones of GABA-immunostained interneurons in cortical neuron cultures. Scale bar = 5 μM. (**B**) The percentage of collapsed growth cones was determined for each condition (300 growth cones per condition, n = 3 experiments, 2-way ANOVA, Bonferonni post-hoc testing, ***p < 0.001).

Growth cone collapse in cultured neuronal cells is widely used for characterization of ephrin/Eph repellent responses^44–46^. Growth cone collapse of wild type (WT) versus NCAM null GABAergic neurons in culture closely correlates with perisomatic synapse densities in WT and NCAM null mice^16,38^. NCAM is required for ephrin-A5-mediated growth cone collapse of GABAergic interneurons *in vitro*^16,38^, but neurocan has not been tested for inhibition of this repellent response. To assess the effect of neurocan on ephrin-A5-induced growth cone collapse, mouse cortical neuron cultures (14 DIV) were treated with or without neurocan (4 nM, 30 min). Neurons were then stimulated with pre-clustered Fc or ephrin-A5-Fc for 30 minutes, and cultures were fixed and immunostained for GABA to identify interneurons. Neuronal growth cones of GABA-positive interneurons were scored as collapsed (bullet-shaped) or not collapsed (fan-shaped) based on morphology as described^16,38^. Ephrin-A5-Fc induced robust growth cone collapse (~55%) in untreated GABA-positive neurons, and this response was fully blocked in cells treated with neurocan (Fig. 7A,B). A fraction of neuronal cells were refractive to ephrin-A5-induced collapse, and may represent a subpopulation that does not express ephrin-A5 receptors, NCAM, and/or required signaling effectors. These results demonstrate that neurocan is capable of blocking ephrin-A5-induced repellent signaling in cortical interneurons. In combination with the effect of chABC on interneuron perisomatic synapse density in slice cultures, this functional assay supports a role for neurocan in inhibitory synapse maturation *in vivo* through inhibition of axonal repellent responses.

## Discussion

Here we identify a previously unknown role for the PNN protein neurocan in disrupting NCAM interaction with EphA3 to terminate repellent responses and limit the number of perisomatic inhibitory synapses made by basket interneurons onto pyramidal neurons in the mouse PFC. Targeting GAG chains of PNN proteins using the enzyme chABC decreased perisomatic synapse numbers in PFC layers 2,3 of organotypic brain slices, highlighting a previously unknown role for PNNs in regulating inhibitory synapse density of PV^+^ interneurons in the PFC. Furthermore, neurocan was able to rescue the observed effects of chABC treatment. The present study further shows that neurocan perturbs NCAM/EphA3 receptor clustering by binding to the same critical determinants in the NCAM Ig2 domain that are required for binding to EphA3. As a consequence, neurocan inhibits EphA3 kinase activation and impairs ephrin-A5-induced growth cone collapse in GABAergic neurons. During postnatal stages of developmental synapse remodeling in the PFC, neurocan accumulated in the extracellular space and was closely associated with excitatory and inhibitory neurons. Neurocan was observed in close apposition with inhibitory perisomatic terminals by light and electron microscopy, but it did not colocalize within synapses.

Removal of the GAG chains of PNN proteins resulted in a decrease in GABAergic perisomatic synapse density in cortical organotypic slice cultures. The current binding studies and previous work suggest that this effect is unlikely to derive from an effect on NCAM binding to other PNN proteins, although experiments knocking down individual PNN components could be informative. A recent study showed that brevican promotes maturation of excitatory synapses contacting the soma of PV^+^ interneurons^47^, thus different PNN components may regulate perisomatic synapses in distinct neuronal cell types.

Polysialylation of NCAM is associated with synaptic plasticity and remodeling of perisomatic synapses^16,48^. Neurocan interacted robustly with non-PSA NCAM isoforms but not with PSA-NCAM. The finding that PSA inhibited NCAM binding to neurocan suggests that polysialylation is a critical modification that allows remodeling during early development, which is later halted by the loss of PSA that occurs during maturation to terminate developmental plasticity. Developmental regulation of NCAM polysialylation may mediate the transition between remodeling of excess basket cell axon terminals and their stabilization at perisomatic synapses by modulating interaction with neurocan. Domain truncation analysis of NCAM mapped the key interaction site to the NCAM Ig2 domain. While the sushi domain of neurocan is essential for binding to L1^40^, this domain was not required for binding to NCAM. An interface involving complementary charged residues in the interacting domains of NCAM (Ig2) and EphA3 (CRD)^38^ was found to mediate NCAM binding to neurocan. Basic residues in NCAM Ig2 (R156 and K162) were essential for interaction of NCAM with both neurocan and EphA3. These residues are positioned within the heparin-binding site of NCAM, which is essential for interaction of NCAM with extracellular matrix HSPGs^49^. Neurocan binding to NCAM at the heparin binding site (which also has affinity for CSPGs^50^) may promote cell-matrix adhesion as a necessary step for synapse stabilization at pyramidal cell soma. This interpretation is compatible with the decreased numbers of perisomatic synapses observed after chABC treatment.

Pulldown experiments with purified proteins demonstrated direct binding of NCAM to neurocan, but not to ephrin-A5 or EphA3. Neurocan interaction with NCAM was strongly dependent on GAG chains, but even the core protein of neurocan retained some binding affinity for NCAM. Neurocan blocked co-clustering of NCAM and EphA3 receptors on processes of cortical interneurons stimulated with the ligand ephrin-A5, suggesting that neurocan binding to NCAM in the plasma membrane displaces EphA3. Neurocan treatment also inhibited ephrin-dependent EphA3 autophosphorylation. As clustering of EphA receptors is essential for kinase activation and downstream signaling at localized sites in the neuronal membrane^41^, the finding that neurocan can destabilize EphA3 clusters reveals an additional level of regulation in modulating ephrin-dependent repellent activity.

In a functional assay of ephrin-dependent signaling, neurocan significantly perturbed ephrin-A5-dependent growth cone collapse. Because NCAM is required for ephrin-A5-dependent growth cone collapse of cortical interneurons^38,51^, results are consistent with the interpretation that neurocan/NCAM interactions impair EphA3 clustering and downstream signaling to inhibit ephrin-A-induced axonal repellent responses. Although growth cone collapse *in vitro* correlates with inhibitory synapse densities observed *in vivo*^16^, the culture assay does not distinguish ephrin-A5-induced growth cone repulsion from elimination of nascent synaptic contacts. To address this question, live imaging of perisomatic synapse formation and elimination *in vivo* could be informative.

**Figure 8:**
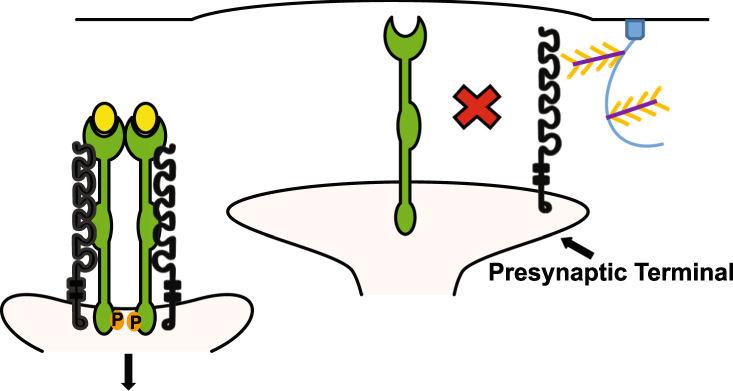
Model of inhibition of NCAM/EphA3 clustering and activation by neurocan. During postnatal remodeling of transient perisomatic synapses made by PV^+^ interneurons onto pyramidal cell soma, ephrin-A5 dimers bind the ligand binding domains (LBD) of an EphA3 dimer. NCAM clusters the EphA3 receptors through binding of the NCAM Ig2 domain to the EphA3 CRD. EphA3 clustering activates tyrosine kinase signaling leading to synapse retraction. With further maturation, neurocan in PNNs engages the Ig2 domain of non-PSA NCAM, inhibiting EphA3 clustering and retraction of inhibitory perisomatic contacts. NCAM is shown in black, EphA3 is green, and ephrin-A5 is yellow. Neurocan core protein is depicted in purple with GAG chains in orange on a PNN scaffold (blue). P = phosphorylation. The illustration was created by modifying images purchased in the PPT Drawing Toolkits-BIOLOGY Bundle from Motifolio, Inc.

This study delineates a novel molecular mechanism in which neurocan disrupts NCAM/EphA3 receptor clustering, EphA3 tyrosine kinase activation, and repellent responses of axon terminals in interneurons in the developing PFC. Results support a model (Fig. 8) in which PSA-NCAM enables NCAM/EphA3 clustering and signaling to eliminate excess presynaptic contacts of PV^+^ interneurons with pyramidal cell soma in early postnatal development. Upon maturation, neurocan in the forming PNNs binds to accumulating non-PSA NCAM, inhibiting ephrinA5/EphA3-mediated repellent responses and stabilizing inhibitory perisomatic synapses (Fig. 8). NCAM and EphA3 are both prominently involved in development of the nervous system, and neurocan is a potential risk factor in schizophrenia^18^ and bipolar disorder^20^. Therefore, understanding how the PNN component neurocan influences the function of NCAM and EphA3 is relevant to both normal development and disease states.

## Materials and Methods

### Mice

Mice were used according to the University of North Carolina Institutional Animal Care and Use Committee (IACUC) policies (AAALAC Institutional Number: #329) and in accordance with NIH guidelines, and all animal protocol procedures were approved by the University of North Carolina IACUC (IACUC ID# 15–243). The *Pvalb*-IRES-Cre line^21^ (JAX No. 008069) was crossed to the Ai9 reporter line^22^ (JAX No. 007909) to label cells with tdTomato after Cre-mediated recombination. Mice were maintained on the C57BL/6 background.

### Immunochemicals and Reagents

Monoclonal antibodies used were directed against NCAM (OB11; Sigma Aldrich), phosphotyrosine (PY99; Santa Cruz Biotechnology), versican (sc-47769, Santa Cruz Biotechnology), C-4-S (MAB2030, Millipore Sigma), and HNK-1 (559048, BD Pharmingen), His tag (MAB050, R&D) and PSA (Ab5324, Millipore). An antibody recognizing a shared epitope of aggrecan and brevican (sc-166951, Santa Cruz Biotechnology) was also used. Polyclonal antibodies were against neurocan (AF5800, R&D), EphA3 (C-19; Santa Cruz Biotechnology), NCAM (H300; Santa Cruz Biotechnology), GAPDH (IMG-3073, Imgenex), GABA (A2052; Sigma and ab17413; Abcam), GAD65 (GAD-6; Developmental Studies Hybridoma Bank), MATH-2 (ab85824; Abcam), and GFP (13970; Abcam). Normal rabbit IgG and goat anti-human IgG were from Jackson ImmunoResearch Laboratories. Secondary antibodies from Life Technologies were anti-mouse Alexa-488, anti-mouse Alexa-555, anti-mouse Alexa-647, anti-rabbit Alexa-555, and anti-rabbit Alexa-647. Other secondary antibodies used were anti-guinea pig Alexa-405 (Sigma Aldrich), anti-rabbit Alexa-405 (Abcam), anti-human IgG-HRP (Sigma-Aldrich), anti-mouse-HRP (Pierce), anti-goat-HRP (Southern Biotech), and donkey anti-rabbit HRP (Sigma-Aldrich). Labeling of PNNs was performed using biotinylated *Wisteria Floribunda* Agglutinin (WFA) (L1766; Sigma) and streptavidin-Alexa-647 (S32357; ThermoFisher). Recombinant ephrin-A5-Fc, human Fc, mouse neurocan, human neurocan, and human tenascin-R (R&D Systems) were also used. Human NCAM-Fc proteins^52^ were purified from transfected HEK293T cell conditioned media using Protein A Sepharose (Pierce). Chondroitinase ABC and penicillinase were purchased from Sigma Aldrich. Endoneuraminidase-N (endo-N), which removes α-2,8 sialic acid chains^53^, was a gift of Urs Rutishauser (Memorial Sloan-Kettering Cancer Center).

### Enzymatic Treatment of Organotypic Brain Slices

Organotypic slice cultures containing the PFC were prepared as in^16^ by sectioning the brain of PV-Cre;Ai9 mice at age P5 in the coronal plane (400 μm). Slices were cultured in Millicell tissue culture inserts in Dulbecco’s Modified Eagle’s Media (DMEM) containing 20% horse serum, 1 mM L-glutamine, 13 mM D-glucose, 1 mM CaCl_2_, 2 mM MgSO_4_, 1 μg/mL insulin, 30 mM HEPES, 5 mM NaHCO_3_, and 0.001% ascorbic acid, which was replaced every 2 days, as described^54^. Slices at 12 days *in vitro* (DIV) were treated with chABC (0.2 U/mL) or control penicillinase (0.2U/ml) for 2 hours as in^55^, followed by 2 rinses with culture media to remove enzyme and another 2 days of culture before fixation on DIV14. Slices were fixed in 4% PFA and stained with antibodies to NeuN to mark neuronal nuclei^56^ (1:400), tdTomato to label perisomatic puncta (1:100), and biotinylated WFA to label PNNs (1:500) followed by detection with streptavidin-Alexa647. A blinded observer scored the average number of perisomatic puncta within 2 μm of NeuN-labeled nuclei from 3 animals per enzymatic treatment condition (30 cells/condition). The efficiency of chABC treatment was verified by imaging WFA labeling compared to the control condition (penicillinase-treated). For rescue experiments, slices were treated with enzyme at DIV 12 for 2 hours, rinsed twice with culture media, and then cultured in media containing no added protein (control untreated), recombinant neurocan (20 μg/ml), or recombinant tenascin-R (20 μg/ml) until fixation on DIV 14. For testing of HNK-1 carbohydrate modification of recombinant tenascin-R, 1 μg of neurocan (negative control) and tenascin-R and 5 μg of P21 mouse brain lysate (positive control) were assessed by immunoblotting for HNK-1 and reprobing for the His tag on recombinant proteins.

### Electron Microscopy and Immunogold labeling

*C57*BL/6 WT mice were anesthetized and perfused transcardially with phosphate buffered saline (PBS) followed by 4% PFA + 0.1% glutaraldehyde in PBS. Brains were postfixed for 2 days in the same fixative, and 50 µm thick coronal sections were cut using a vibratome. Pre-embedding immunogold labeling with silver enhancement was performed for neurocan. To quench glutaraldehyde, sections were pretreated with 1% NaBH_4_ in PBS and then 3% H_2_O_2_ in PBS, followed by blocking in 10% normal donkey serum. Sections were incubated overnight in primary antibody diluted at 1:500–1:2000 in PBS, rinsed, briefly re-blocked in 2% normal donkey serum, and incubated for 2 hours in biotinylated donkey anti-sheep secondary (Jackson ImmunoResearch 713–065–147). Samples were rinsed and incubated 1 hour in streptavidin-nanogold (Nanoprobes 2016) at 1:100 in 1% normal donkey serum/PBS. Sections were stabilized in 2% glutaraldehyde/PBS for 30′ and then rinsed in PBS followed by 0.05 M sodium acetate. After transfer to new vials, sections were silver-enhanced for 7′ using HQ silver enhancement kit (Nanoprobes 2012), rinsed with 0.05 M sodium acetate, and moved to phosphate buffer, pH 6.8. Sections for electron microscopy were then post-fixed 45′ in 0.5% osmium tetroxide in phosphate buffer, pH 6.8, rinsed, and placed in 0.1 M maleate buffer, pH 6, followed by 45′ in 1% uranyl acetate/MB. Sections were dehydrated through a graded ethanol series ending with 2 changes of 100% ethanol. Resin infiltration was 30′ in 1:1 ethanol/resin, 30′ 1:3 ethanol/resin, then 2 changes in pure resin (Low Viscosity embedding kit—from Dr. Spurr, Electron Microscopy Sciences 14300). Infiltrated sections were sandwiched between 2 strips of ACLAR film (between 2 glass slides for support) and polymerized for 36–48 hours in an oven. Small (1 × 1 mm) areas of interest from neocortex were cut out and glued onto a support block. The tissue was trimmed and sectioned (50–60 nm) (Leica Ultracut R) and sections collected on 300 mesh nickel or copper grids. Tissue sections were then counterstained with uranyl acetate followed by Sato’s lead and examined with a Tecnai 12 electron microscope.

### Immunoprecipitation and Pull-Down Assays

For coimmunoprecipitation of neurocan with NCAM140, NCAM mutants, or EphA3, HEK293T cells (cultured in DMEM, 10% fetal bovine serum) were transfected with EphA3 or NCAM140 cDNA (in pcDNA3.1 vectors) using Lipofectamine 2000 (Invitrogen). Proteins from cell lysates (1 mg) in RIPA buffer (20 mM Tris pH 7.0, 0.15 M NaCl, 5 mM ethylenediaminetetraacetic acid (EDTA), 1 mM EGTA, 1% NP-40, 1% deoxycholate, 0.1% sodium dodecyl sulfate (SDS), 200 μM Na_3_VO_4_, 10 mM NaF, 1 × protease inhibitor (Sigma)) were prepared as described^57^, and proteins were precipitated using antibodies against EphA3 (C-19, Santa-Cruz), or NCAM (H-300 Santa-Cruz), with Protein A/G agarose beads (Thermo-Fisher). Co-immunoprecipitation from brain was performed using 1 mg of lysate in RIPA buffer. In some experiments, brain extracts were treated for 1 hr with endo-N (40 U) on ice prior to immunoprecipitation to remove PSA^16^. Protein complexes were separated by SDS-polyacrylamide gel electrophoresis (SDS-PAGE), immunoblotted using antibodies to versican, aggrecan/brevican, neurocan, EphA3, NCAM, or phosphotyrosine, and detected with HRP-conjugated secondary antibodies. Pulldown analyses were performed by incubating 1 μg of neurocan (human or mouse) with Fc protein (1 μg) (control Fc, NCAM-EC-Fc, NCAM Ig1-3, NCAM Ig1-2, NCAM Ig2, NCAM Ig1, or ephrin-A5-Fc) in TBS for 1 hr at 37 °C. Protein A/G Sepharose beads were used to pull down Fc proteins for detection by immunoblotting. For detection of EphA3 autophosphorylation, HEK293T cells were cotransfected with NCAM-140 and EphA3 cDNA. Transfected cells were pre-treated with neurocan (20 nM) for 30 min prior to treatment with preclustered ephrin-A5-Fc or control Fc^58^ for 10 min, then lysed in RIPA buffer. EphA3 was immunoprecipitated, and tyrosine phosphorylation was detected with anti-PY99.

### Competitive Binding Assay

HEK293T cells were transfected with NCAM-140 and EphA3. Recombinant neurocan was treated with 0.1 U/microgram chABC (Sigma) at 37 °C for 1.5 hr or incubated with no enzyme (as a control), followed by heat inactivation of enzyme at 100 °C for 10 min. Efficacy of chABC treatment was assessed by immunoblotting. Cells were treated with neurocan (20 nM), chABC treated neurocan (20 nM), or no neurocan (control) for 30 min. Cells were lysed and NCAM immunoprecipitated, and bound EphA3 and neurocan were detected by immunoblotting.

### Immunostaining and Co-localization Analysis

For cortical neuron cultures, embryonic day 0.5 (E0.5) was defined as the plug date and cortical neuron cultures prepared at E15.5. Dissociated cortical neurons from WT mice were plated onto poly-D-lysine- and laminin coated Lab-Tek II chamber slides (1.5 × 10^5^ cells/well) as described^23,57^. At 10 DIV, cells were pretreated with or without neurocan (4 nM) and then stimulated with 1 μg/mL preclustered Fc or ephrin-A5-Fc for 30 min. Cells were fixed in 4% paraformaldehyde and blocked/permeabilized in 0.5% TritonX-100/PBS with 10% horse serum. Slides were incubated in primary antibodies against NCAM, EphA3, and GABA overnight at 4 °C. Secondary antibodies (Alexa 405, Alexa 555, Alexa 647) were added for 1 hr and mounted using SlowFade (Life Technologies). Confocal images were obtained with Zeiss LSM700 and LSM710 microscopes using a Plan-Apochromat 63 × 1.4 numerical aperture objective with 2X optical zoom using Zeiss Zen software. Only GABA-positive cells were imaged for analysis. Co-localization of NCAM and EphA3 was analyzed using the ImageJ plugins Colocalization_Test and Colocalization_Threshold as previously^38^. Co-localization was expressed as R-Total (the Pearson correlation coefficient, which varies between −1 and 1). Values were calculated for pixels above a threshold level determined by the regression algorithm contained in the “Co-localization_Threshold” macro. For each condition, an average from measurements of at least 30 images was reported.

### Growth Cone Collapse Assay

Dissociated cortical neuron cultures were generated from WT mice as described above. At 10 DIV, cells were pre-treated with or without neurocan (4 nM) and then with preclustered ephrin-A5-Fc or human Fc (1 μg/mL) for 30 min, fixed, and growth cones visualized by immunofluorescence staining of GABA^16,38^. Growth cones were scored as collapsed by bullet-shaped morphology or non-collapsed by spread morphology by an observer blinded to treatment, and the percentage of collapsed growth cones of GABA-positive neurons was compared (10 fields/well; ≥2 wells/experiment; ≥300 growth cones/condition).

### Data Availability

The datasets generated and analyzed as part of the current study are available from the corresponding author upon request.
